# Supplementary figures and images for: Identification of drug-like molecules targeting the ATPase activity of dynamin-like EHD4
Source: PLoS One. 2024 Jul 29;19(7):e0302704. doi: 10.1371/journal.pone.0302704 (PMC11285977; doi:10.1371/journal.pone.0302704)

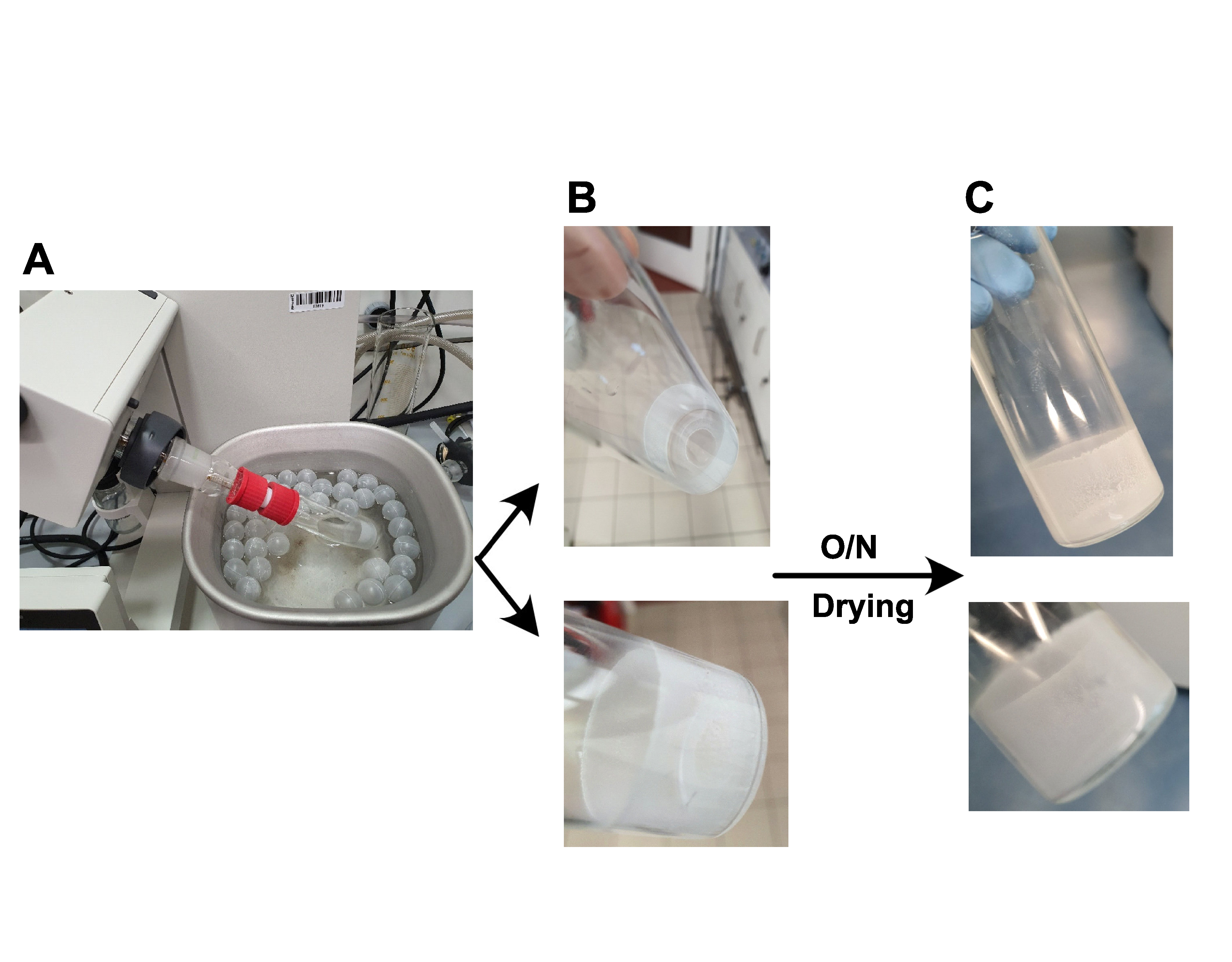

Supplement: S1 Fig — A Overview of the experimental setup. Nitrogen gas instead of vacuum was used to evaporate the chloroform/methanol mix from the lipid solution. For this, the vacuum port was removed and a 50 ml serological pipette attached to a nitrogen gas tubing into the glass vial (VWR 548–0156). 500 μl of DOPS (Avanti Polar Lipids) were mixed with 6 ml of a chloroform/methanol mixture (3:1 v/v) in the previously mentioned glass vial. This vial was attached to a Rotavapor R-300 (Buchi) with the bottom part of the vial dipping into the heating bath kept at 25°C. A gentle nitrogen stream was introduced into the vial and adapted to not observe ripples on the surface of the solution. The rotation speed was set to 175 rpm. B Approximately 25 min later, a regular lipid monolayer formed on the glass vial after removal of chloroform/methanol mixture using the Rota vapor. C Same lipids after overnight (O/N) drying. (TIF) [file pone.0302704.s001.tif]

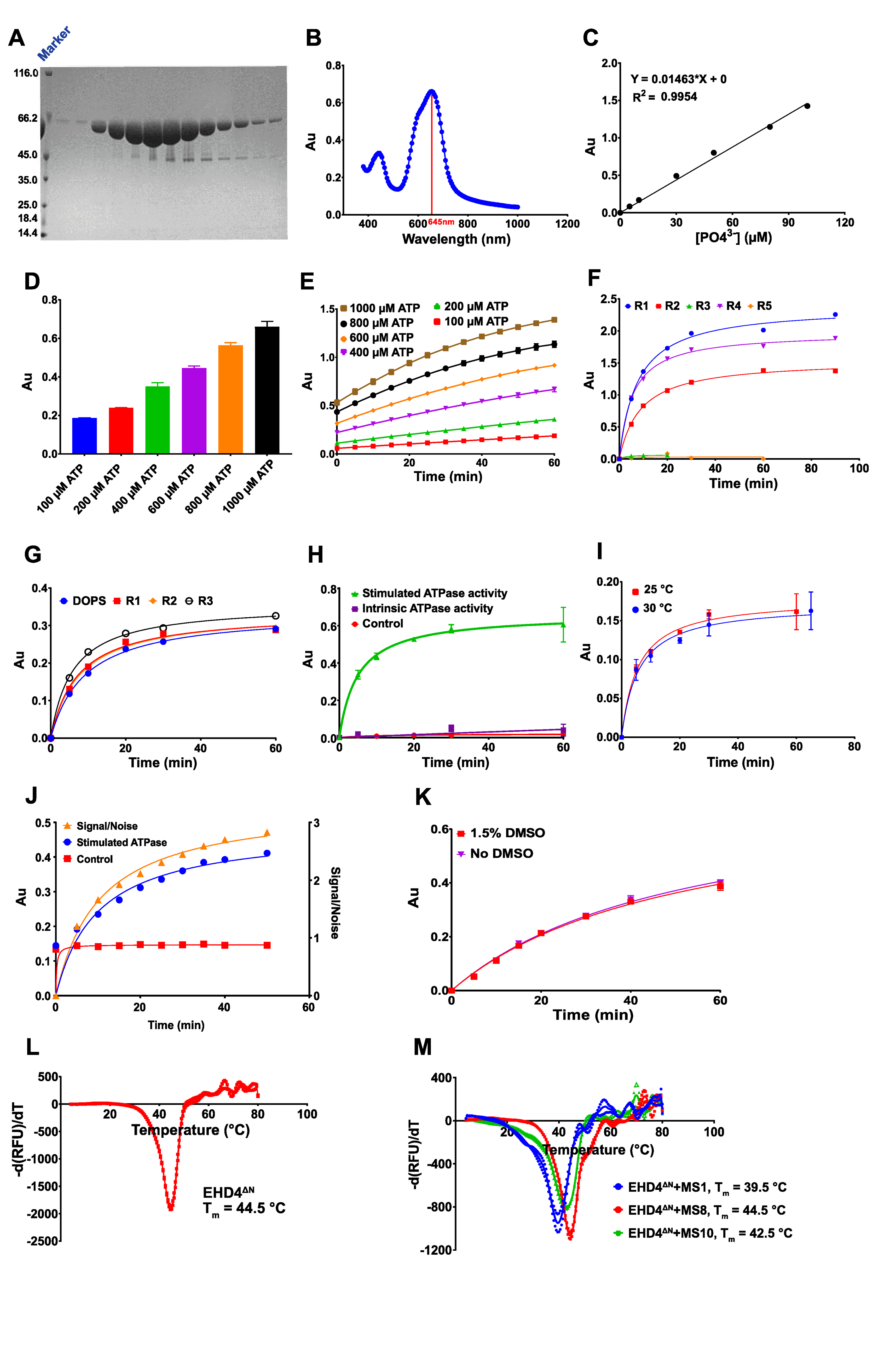

Supplement: S2 Fig — A Coomassie-stained SDS-PAGE gel showing fractions of the final gel filtration peak of the EHD4ΔN purification. B Absorption maxima of the colorimetric complex formed between the MLG dye, molybdate and orthophosphate. AU–arbitrary units. C Standard curve of orthophosphate in the MLG assay. The determined fit parameters of the curve are shown on the top left. D Background signal from ATP possibly derived from phosphate contaminations is directly proportional to the ATP concentration in the assay. E ATP hydrolysis in the absence of EHD4 ΔN and DOPS in the acidic environment of the MLG dye over time. Note that higher ATP concentration leads to a higher background signal. F ATPase activity of EHD4ΔN at 25°C at 2 μM EHD4ΔN, 200 μM ATP and 300 μg/ml liposomes composed of natural PS in assay buffer was detected by the MLG assay, but it was not reproducible. R1-R5 represents different repetitions of the EHD4 assay under supposedly identical conditions. G ATPase assay of EHD4ΔN at 4 μM EHD4ΔN, 30 μM ATP and 500 μg/ml synthetic DOPS liposomes in assay buffer were conducted at 25°C. R1, R2 and R3 represent three independent preparations of DOPS liposomes from different batches, resulting in similar activities. H MLG-based ATPase assay at 4 μM EHD4ΔN, 30 μM ATP at 25°C in the absence (non-stimulated) or presence of 500 μg/ml DOPS (stimulated). Control represents the assay without EHD4ΔN. I ATPase assay comparison at 30°C and at 25°C at a concentration of 20 μM ATP and 4 μM EHD4ΔN and 500 μg/ml DOPS liposomes in assay buffer. J MLG-based ATPase assay at 200 nM EHD4ΔN, 50 μg/ml DOPS and 30 μM ATP at 25°C without background subtraction. Signal:noise is plotted on the right y-axis. Control represents the assay without EHD4ΔN. K Inclusions of 1.5% DMSO had no effect on EHD4ΔN enzymatic activity in the presence of liposomes, rendering the assay compatible with the HTS setup. L Thermal shift assay of EHD4ΔN at a protein concentration of 5 μM to determine the melting temperature of the [file pone.0302704.s002.tif]

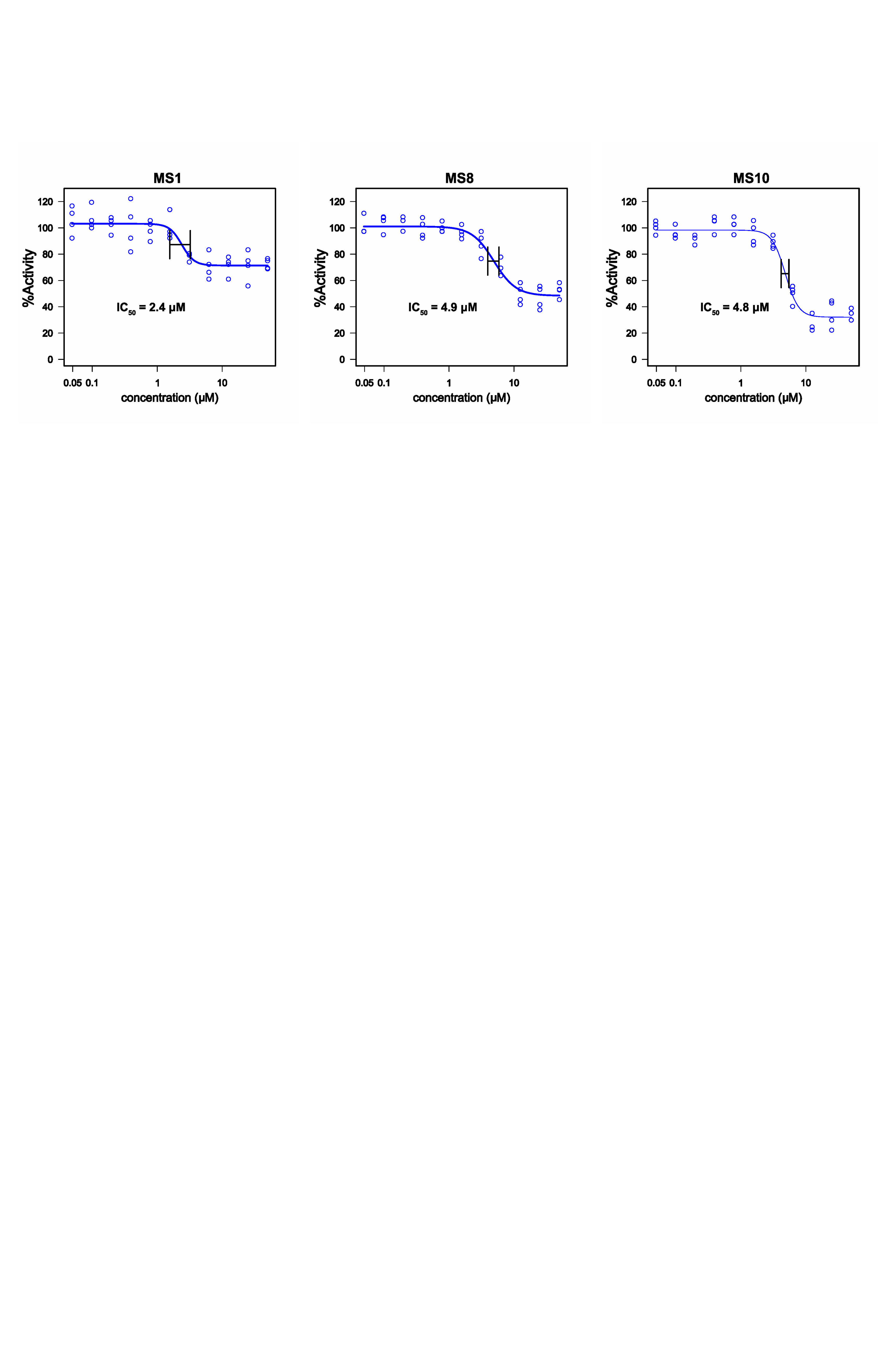

Supplement: S3 Fig — IC50 values for the purchased primary hits were similar to the previously determined IC50 values of the compounds from the drug library. Repurchased MS1 showed an IC50 value of 2.4 μM (vs 0.92 μM with the compound from the drug library), MS8 an IC50 value of 4.9 μM (vs 3.8 μM with the compound from the drug library), MS10 an IC50 value of 4.8 μM (vs 2.9 μM with the compound from the drug library). (TIF) [file pone.0302704.s003.tif]

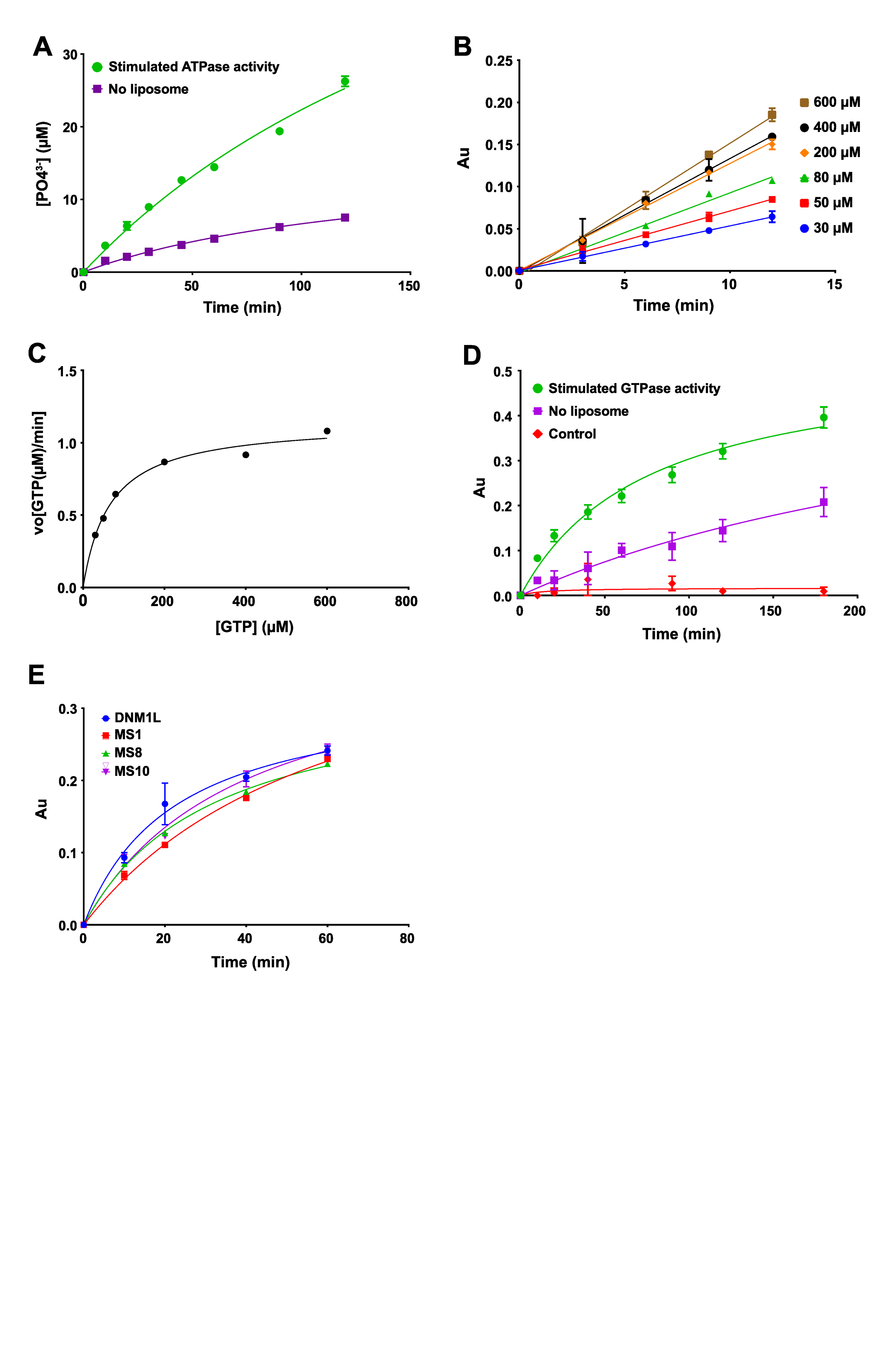

Supplement: S4 Fig — A EHD2 ATPase activity by an HPLC-based method. Assay conditions were 5 μM EHD2, 50 μM ATP and 1 mg/ml Folch liposomes at 30°C. Folch liposomes were used here as the HPLC-based assay is compatible with Folch liposomes. B Initial velocities of DNM1L GTPase activity at 300 nM DNM1L, and, 500 μg/ml synthetic DOPS, T = 25°C and at different GTP concentrations were determined by a linear fit. C Km was determined by first calculating the amount of hydrolyzed GTP in (B) using the standard curve reported in S2C Fig and then plotting the initial rates of the reactions versus the substrate concentration. The kinetic parameters for DNM1L are Km = (66 ± 9) μM, kcat = (3.81 ± 0.15) 1/min, vmax = 1.15 μmoles GTP/min. D GTPase assay monitoring DNM1L activity were done with the final optimized parameters, which were 300 nM DNM1L, 40 μM GTP, 200 μg/ml synthetic DOPS liposomes, T = 25°C in 20 mM HEPES (pH 7.5), 150 mM KCl, 0.5 mM MgCl2. Z´ and SNR were 0.72 and 2.1 at 20 min for the stimulated GTPase activity. E One-hour time course of the MLG-based enzymatic GTPase assay in the presence of 10 μM inhibitor. DNM1L (blue curve) represents the positive control. The data points except in C represent the mean of two independent experiments and the error bar signifies the range of the fit. When the range is smaller than the size of the data point, the error bar is not displayed. (TIF) [file pone.0302704.s004.tif]

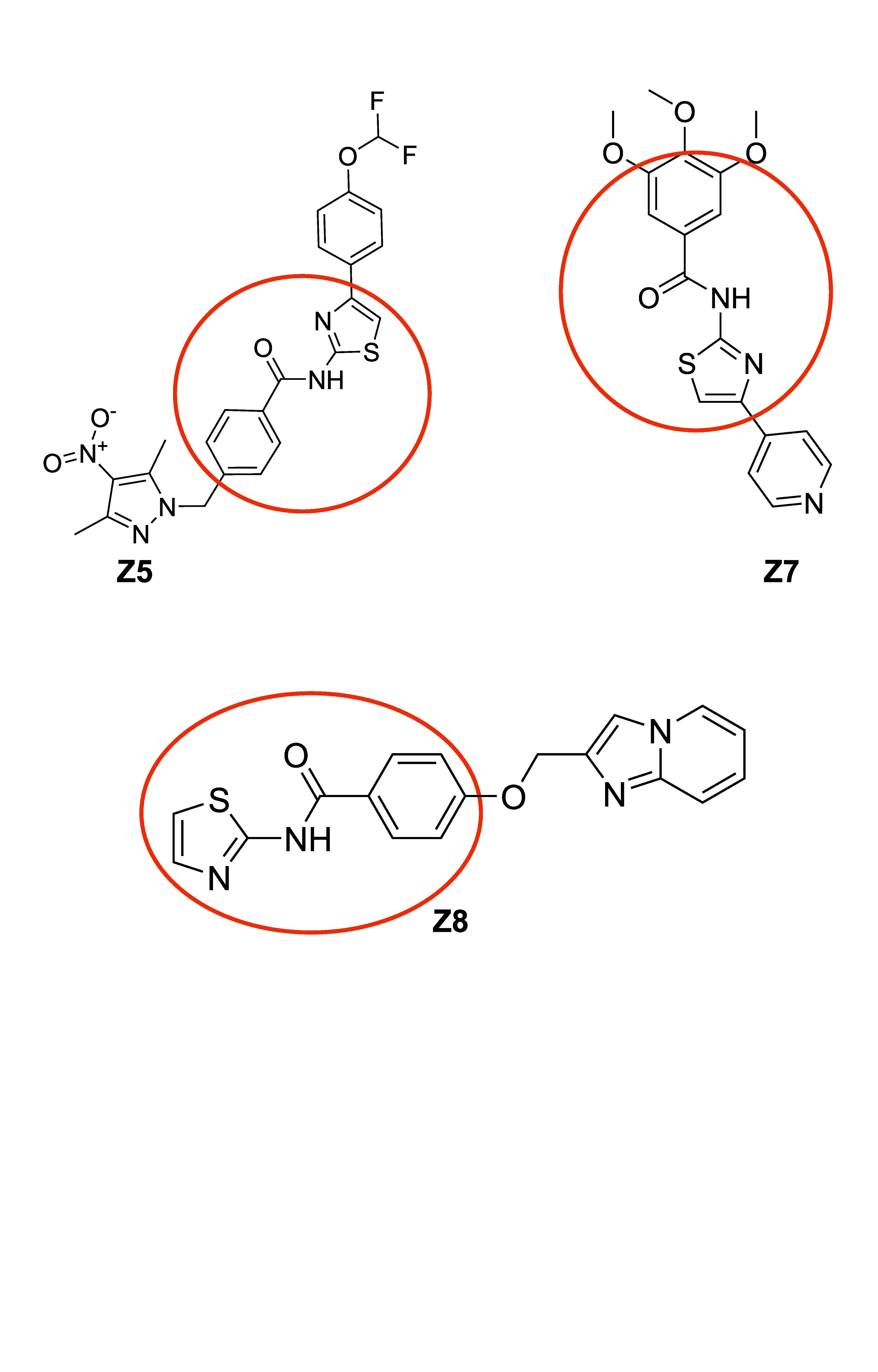

Supplement: S5 Fig — The three compounds that showed inhibition towards EHD4ΔN at 50 μM inhibitor concentration by 20% or more in the MLG assay, among the 55 compounds from the substructure search in our SAR study. Z5, Z7 and Z8 inhibited EHD4ΔN enzymatic activity by 21%, 44% and 38% respectively. The N-(thiazol-2-yl)benzamide moiety, used for our chemical search is encircled in red. (TIF) [file pone.0302704.s005.tif]
