# Supplementary material for: Identification of drug-like molecules targeting the ATPase activity of dynamin-like EHD4
Source: PLoS One. 2024 Jul 29;19(7):e0302704. doi: 10.1371/journal.pone.0302704 (PMC11285977; doi:10.1371/journal.pone.0302704)
Supplement: S1 Table — (PDF) [file pone.0302704.s006.pdf]

105229	inactive	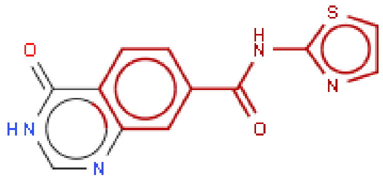
106136	inactive	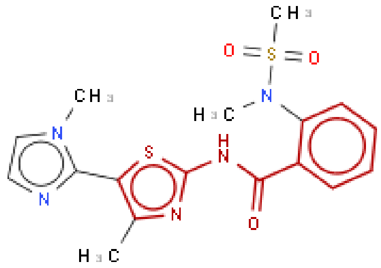
106268	inactive	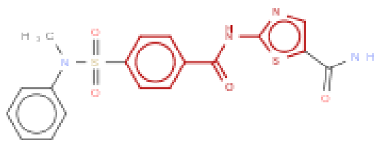
106299	inactive	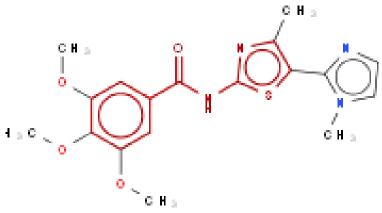

106333	inactive	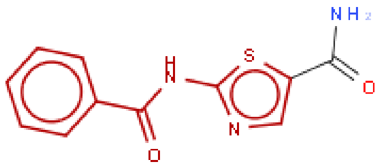
106647	active / Z7	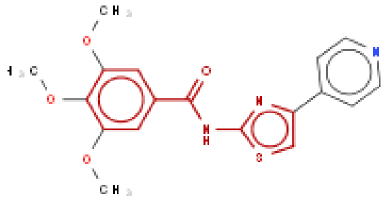
106808	active / Z8	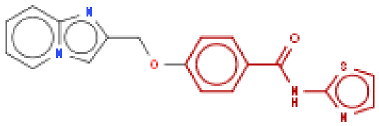
110278	inactive	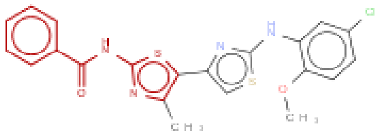

110477	inactive	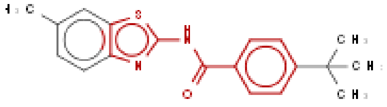
110723	inactive	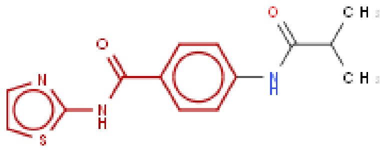
110741	inactive	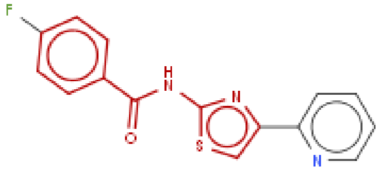
110880	active / MS8	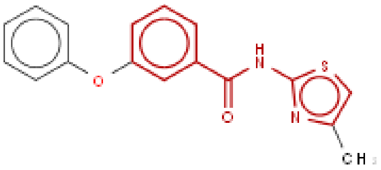

201598 inactive

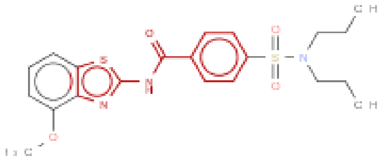

201599 inactive

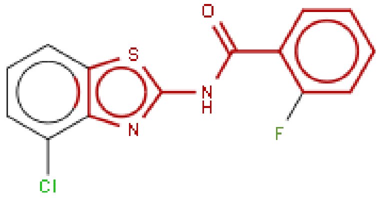

201820 inactive

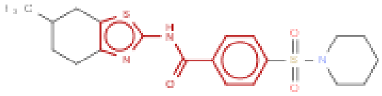

201821 inactive

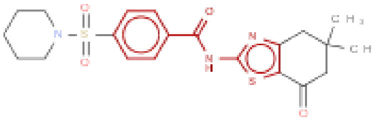

201822	inactive	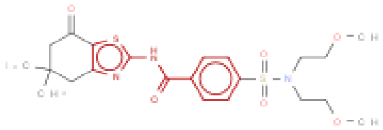
202103	inactive	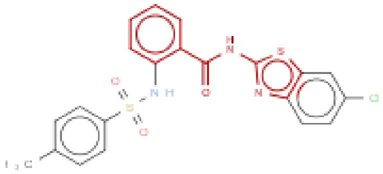
202486	inactive	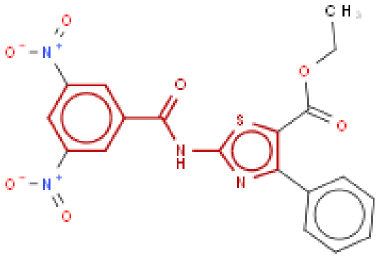
202487	inactive	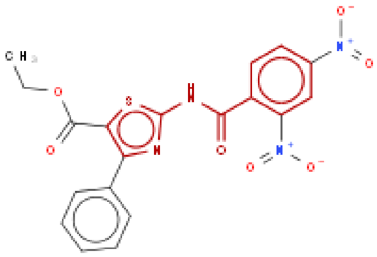

203982	inactive	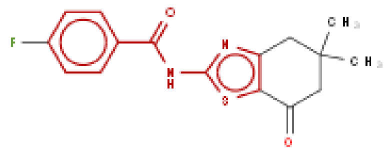
203983	inactive	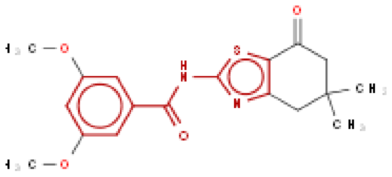
203984	inactive	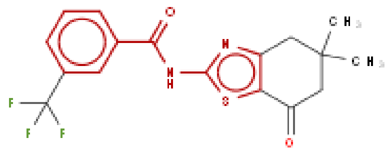
203985	inactive	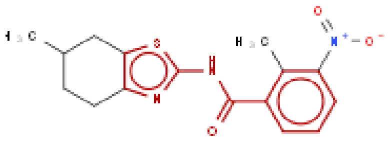

203986	inactive	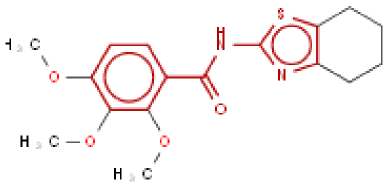
203987	inactive	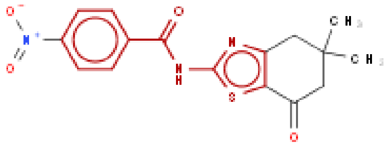
203988	inactive	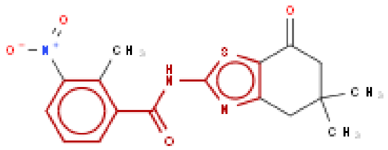
203989	inactive	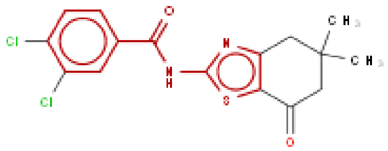

203990	inactive	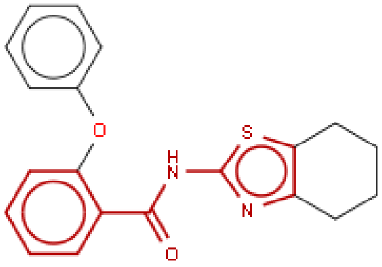
203991	inactive	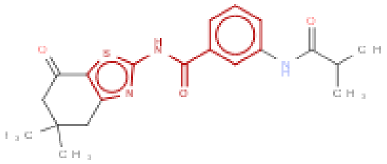
203992	inactive	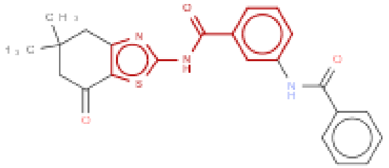
203993	inactive	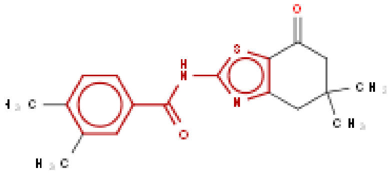

203994	inactive	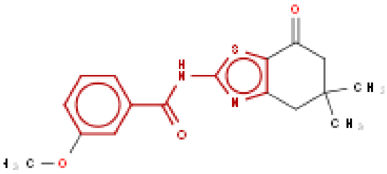
203995	inactive	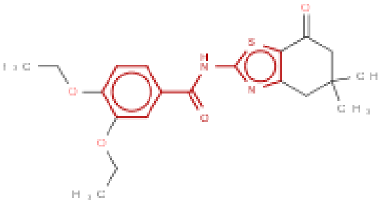
203996	inactive	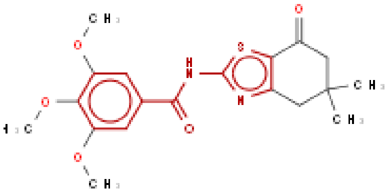
203997	inactive	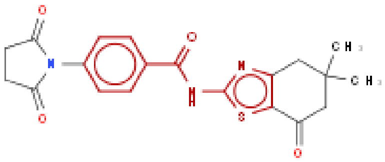

205107	inactive	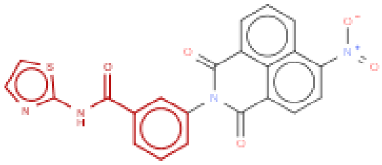
209726	inactive	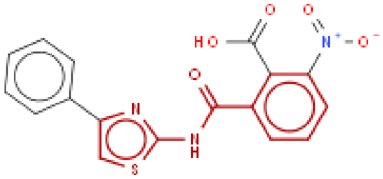
300963	inactive	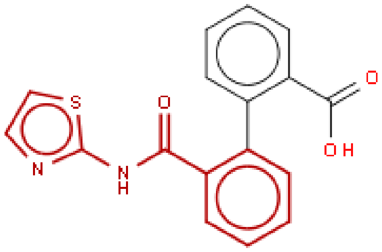
301620	inactive	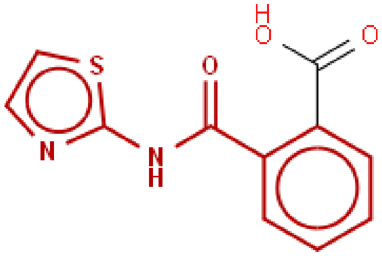

303331	inactive	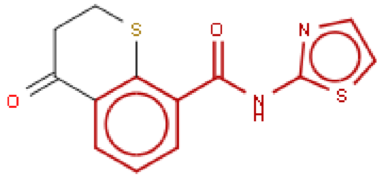
303712	inactive	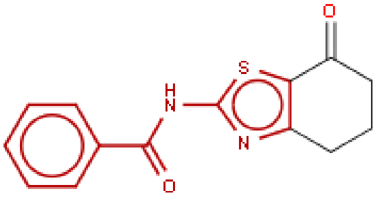
400087	inactive	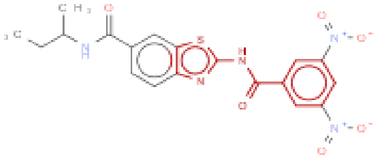
401885	active / Z5	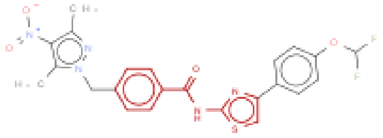

402169	inactive	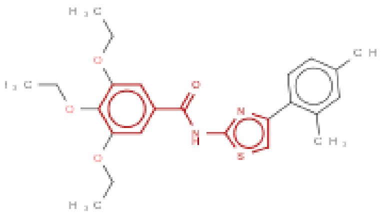
402685	inactive	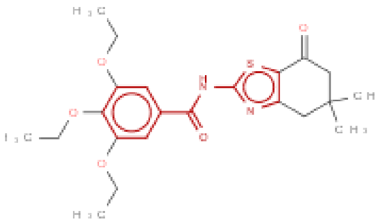
402868	inactive	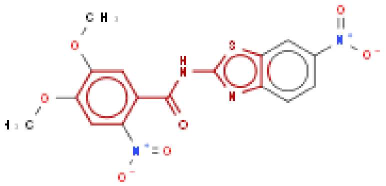
402870	inactive	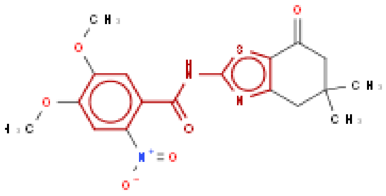

402911	inactive	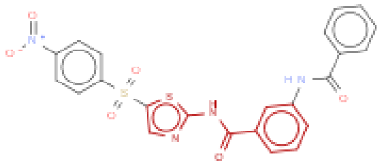
402913	inactive	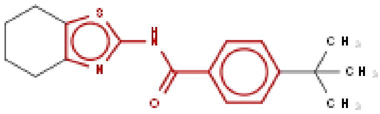
402970	inactive	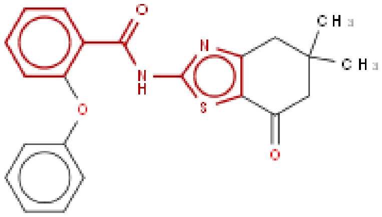
402980	inactive	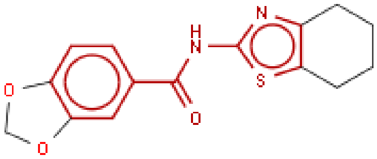

402981	inactive	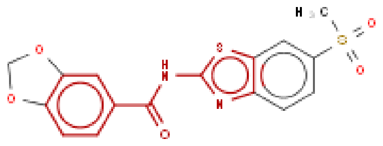
403511	inactive	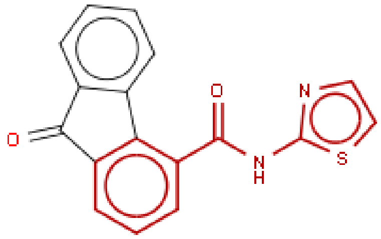
405759	inactive	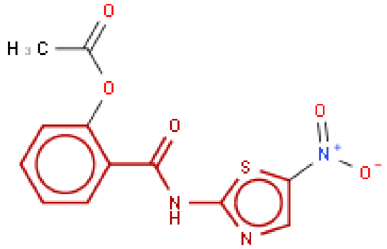
