# Supplementary material for: Identification of drug-like molecules targeting the ATPase activity of dynamin-like EHD4
Source: PLoS One. 2024 Jul 29;19(7):e0302704. doi: 10.1371/journal.pone.0302704 (PMC11285977; doi:10.1371/journal.pone.0302704)
Supplement: S1 Raw images — (PDF) [file pone.0302704.s007.pdf]

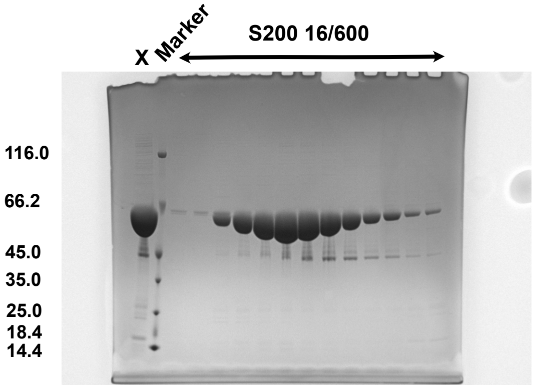

S1 raw image
Raw image of coomassie-stained SDS-PAGE gel showing individual fractions of the final gel filtration (S200 16/600) peak for the EHD4$^{\Delta N}$ purification. S2_Fig. A was generated from this original image.
